# Impact of pre-segmented regions on CT-based evaluation of the Peritoneal Cancer Index: A reader study

**DOI:** 10.1371/journal.pone.0349606

**Published:** 2026-06-01

**Authors:** Lotte J. S. Fleurkens-Ewals, Marion W. Tops-Welten, Anna F. van Herwijnen, Pieter C. Gort, Cris H. B. Claessens, Ellen S. R. Duijsings, Irene E. G. van Hellemond, Jurgen M. J. Piek, Ignace H. J. T. De Hingh, Max J. Lahaye, Misha D. P. Luyer, Joost Nederend

**Affiliations:** 1 Department of Surgical Oncology, Catharina Cancer Institute, Catharina Hospital Eindhoven, Eindhoven, the Netherlands; 2 Department of Radiology, Catharina Cancer Institute, Catharina Hospital Eindhoven, Eindhoven, the Netherlands; 3 Department of Medical Oncology, Catharina Cancer Institute, Catharina Hospital Eindhoven, Eindhoven, the Netherlands; 4 GROW School for Oncology and Developmental Reproduction, Maastricht University, Maastricht, the Netherlands; 5 Department of Electrical Engineering, Eindhoven University of Technology, Eindhoven, the Netherlands; 6 Department of Obstetrics and Gynaecology, Catharina Cancer Institute, Catharina Hospital Eindhoven, Eindhoven, the Netherlands; 7 Department of Radiology, The Netherlands Cancer Institute, Amsterdam, the Netherlands; University of Pisa, ITALY

## Abstract

**Objectives:**

Accurate assessment of peritoneal tumour burden is crucial for treatment selection and prognosis. The Peritoneal Cancer Index (PCI), traditionally assessed by laparoscopy, is increasingly applied to imaging. This study investigates whether pre-segmented radiological PCI (rPCI) regions on CT improve interobserver variability, diagnostic accuracy, and user experience.

**Materials and methods:**

This prospective, comparative reader study included nine contrast-enhanced CT scans from patients with peritoneal metastases of ovarian, gastric, or colorectal origin. Radiologists, surgeons, and gynaecologists independently scored rPCI on two scans: one with and one without pre-segmented rPCI regions shown as colour overlays. Interobserver variability, mean absolute error compared with surgical PCI (sPCI), and reader confidence were compared between both conditions. User experience was evaluated through structured feedback.

**Results:**

In total, 82 clinicians from 19 countries participated in this study. Each CT was assessed 8–10 times with and without overlaid segmentations. Pre-segmented region overlays did not significantly improve interobserver variability (p = 0.123), PCI accuracy (mean absolute error, p = 0.374), or reader confidence (p = 0.593). However, with use of segmentations, rPCI values were closer to sPCI in 7 of 9 scans. In regions 9–12, the proportion of participants reporting moderate or high confidence increased from 48–53% without overlays to 59–63% with overlays. User feedback was positive, with 83% of participants reporting the tool as useful, 82% as easy to use, and 71% indicating they would use it in clinical practice.

**Conclusion:**

Pre-segmented region overlays for CT-based rPCI assessment were positively received by clinicians. Although no statistically significant improvements in interobserver variability or accuracy were observed, the findings of this exploratory study support further evaluation in larger studies to determine their clinical value.

## Introduction

Peritoneal cancer is a malignant disease affecting the peritoneum, the membrane covering the abdominal cavity and its organs [1]. Most cases arise from metastatic spread of primary gastric, colorectal, or ovarian cancer, although in rare cases primary peritoneal cancer may also occur [2]. Peritoneal cancer is associated with poor survival rates, which has driven the development of novel intraperitoneal treatment strategies, several of which are currently under investigation [3,4].

Accurate assessment of peritoneal tumour burden is essential for guiding treatment selection and determining prognosis [5–7]. The Peritoneal Cancer Index (PCI) is currently considered the gold standard for quantifying the extent of peritoneal disease [5]. It is calculated by dividing the abdomen into 13 regions, each scored from 0 and 3 according to the largest peritoneal nodule, resulting in a total PCI from 0 to 39 [8]. This score reflects overall tumour burden and strongly correlates with survival and the likelihood of residual disease after cytoreductive surgery, making it an essential tool for treatment selection [6,9,10].

Traditionally, PCI is assessed by diagnostic laparoscopy (DLS). In clinical practice, PCI is also increasingly assessed using imaging, mostly Computed Tomography (CT) or Magnetic Resonance Imaging (MRI). This imaging-based assessment, referred to as radiological PCI (rPCI), has been shown to correlate with surgical outcomes and survival, and can identify patients unsuitable for surgery due to disease extent [11]. In addition, imaging may provide complementary value to DLS by aiding preoperative planning and intraoperative guidance. Certain anatomical regions, such as the lesser sac or subphrenic spaces, may be visualized more reliably on cross-sectional imaging than during laparoscopy [12,13]. Nevertheless, small tumour deposits often remain difficult to detect on imaging, requiring considerable radiological expertise and leading to high interobserver variability [14–16].

An important challenge is that the original 13-region definitions by Sugarbaker et al. lack explicit anatomical boundaries for rPCI assessment, reducing the reproducibility of imaging interpretation. To address this, a recent Delphi study introduced standardized rPCI definitions to improve the consistency in imaging-based PCI scoring [17]. Recent studies have also explored AI and radiomics approaches for the evaluation of peritoneal metastases on imaging. However, most existing models focus on predicting the presence of metastases or broad PCI categories, often based on primary tumour characteristics rather than direct region-based assessment, which limits their clinical applicability for staging and treatment selection [18]. To support adoption of the standardized rPCI definitions into clinical practice, an artificial intelligence (AI) model is currently being developed to automatically segment the 13 PCI regions on imaging. These pre-segmented regions may help radiologists in identifying and scoring peritoneal lesions in a more structured and consistent manner.

This exploratory study aimed to evaluate the impact of using pre-segmented regions on CT for rPCI assessment, focussing on interobserver variability, accuracy in rPCI estimation, and participants’ perceptions of usability and added clinical value.

## Methods

This was a prospective, comparative reader study in which clinicians evaluated CT scans of patients with peritoneal metastases. The study was approved by the Institutional Review Board of the Catharina Hospital Eindhoven (nWMO-2022.142) and conducted in accordance with the ethical guidelines of the Declaration of Helsinki. Clinicians provided written informed consent through an online questionnaire and participated voluntarily by completing a questionnaire in which they evaluated anonymized CT scans. As the patients were deceased, patients’ informed consent was waived. No minors were involved.

### CT scans

Nine contrast-enhanced CT scans from patients with peritoneal metastases were used in this study, with three scans each representing primary ovarian, gastric, and colorectal cancer, acquired between September 2017 and July 2023. All patients underwent both CT and surgical PCI (sPCI) assessment within a six-week interval without any intervening treatment, allowing comparison of rPCI and sPCI. For each cancer type, one case representing a low (<10), moderate (10–20), and high sPCI (>20) was included. The slice thickness was 3 mm, except for the gastric cancer case with low PCI (axial 3.75 mm, coronal 5 mm). The 13 PCI regions were manually segmented for each scan by three researchers, based on the rPCI definitions [17]. Segmentations were performed using 3D Slicer (v5.8.1) [19]. All segmentations were performed in an iterative process under the supervision of two radiologists with subspecialty expertise in oncologic and abdominal imaging (J.N. and M.L., each with >12 years of experience as a radiologist), and were reviewed and refined in consensus. To ensure complete coverage and minimize the risk of missing relevant voxels, each segmented region was dilated by 2 mm in all directions where feasible, with overlapping voxels assigned to the closest region.

### Participants

Radiologists, surgeons, and gynaecologists were recruited between 25 September 2024 and 21 May 2025 via international and national professional networks: the European Society for Gastrointestinal & Abdominal Radiology (ESGAR), the Peritoneal Surface Oncology Group International (PSOGI), and Dutch societies of abdominal radiologists and working group of gynaecological oncologists. Recruitment methods included emails, LinkedIn posts, and conference presentations. Additionally, participants were invited to refer colleagues with relevant expertise, who were subsequently contacted and invited to participate. Clinicians completed a short questionnaire to register, providing their contact details, medical specialty, years of experience evaluating peritoneal cancer on imaging, and rPCI evaluation frequency (daily, weekly, monthly, yearly, or never). Based on this information, we aimed for a balanced distribution of reviewer experience across scans to enable representative comparison between groups. In line with clinical practice, surgeons were assigned only to gastric and colorectal cases, gynaecologists to ovarian cancer cases, and radiologists could be assigned to all cancer types.

### Study procedures

**Fig 1:**
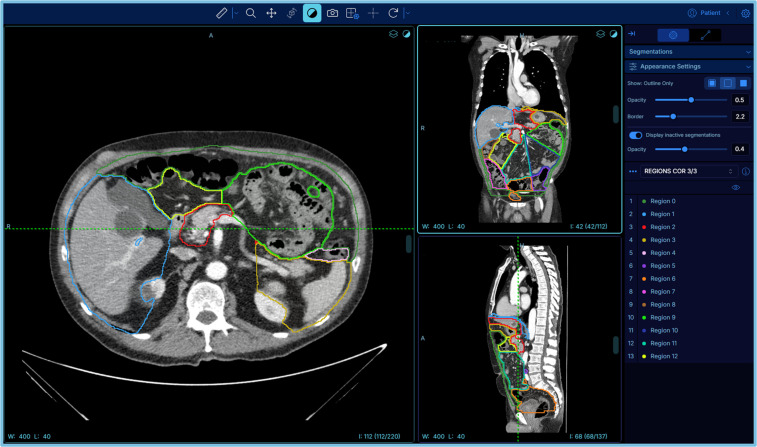
Screenshot of multiplanar reconstructions of a CT scan during evaluation with pre-segmented PCI regions, visualized using coloured boundaries. The image includes a screenshot of the OHIF Viewer interface, reproduced under the MIT License (Copyright © 2018 Open Health Imaging Foundation).

Each participant evaluated PCI of two different CT scans: one without region overlays and another with the 13 rPCI regions visualized as colour overlays (Fig 1). Instructions and links to the scans and questionnaires were distributed via email. Scans were accessed using a self-optimized instance of OHIF Viewer (v3.10.0-beta.69), which allowed viewing in all anatomical planes [20]. The technical possibilities for settings were explained to the readers, including how to measure nodule sizes, zoom, move, and adjust contrast settings. Furthermore, when assessing the scan with the overlaid segmentations, appearance of the segmentations could be changed according to the user’s preference, such as the opacity and border thickness.

Within the online questionnaire, participants scored PCI for all 13 regions for each allocated scan. For each region, they rated their confidence in the accuracy of their assessment using a 5-point scale (not confident at all, slightly confident, somewhat confident, moderately confident, or extremely confident), and indicated the imaging plane(s) used to measure the lesion size. They also rated their statements regarding the usefulness, usability, and impact of the segmented regions on their PCI assessment, including effects on accuracy, efficiency, and potential use in clinical practice, and could provide additional explanations for their responses.

### Analyses

The interobserver variability was analysed by comparing the interquartile ranges (IQRs) of the total PCI scores per scan, in a paired manner with and without the use of the pre-segmented regions. This was visualized using boxplots.

In the same boxplots used to assess interobserver variability, the sPCI was specified to allow a visual comparison of the participants’ rPCI accuracy relative to the gold standard. To explore whether participants’ background influenced PCI assessment, scores were descriptively stratified by medical specialty (radiologist/radiology resident, surgeon, gynaecologist/gynaecological surgeon) and experience in evaluating peritoneal metastases on imaging. The results were visualized as scatterplots, displaying individual participant rPCI scores in relation to sPCI, separately for each scan, assessed with and without the pre-segmented regions. Furthermore, the accuracy was quantitatively evaluated by calculating the Mean Absolute Error (MAE) between rPCI and sPCI scores for each scan. The MAEs were assessed per scan and compared pairwise between evaluations with and without region segmentations.

The mean confidence score of all regions was computed for each evaluated scan. Subsequently, the mean of these average confidence scores per scan was computed for evaluations with and without the use of the pre-segmented regions, and compared in a paired manner. Furthermore, we assessed whether confidence scores differed per region between evaluations with and without using the pre-segmented regions. For each region, the mean confidence per scan was calculated and averaged across all nine scans, and these values were compared in a paired manner between the evaluations performed with and without the pre-segmented regions. Furthermore, the confidence distributions per region were visualized in a bar graph.

Statistical analyses were performed using IBM SPSS Statistics for Windows, version 29 (IBM Corp., Armonk, N.Y., USA). Differences in IQRs and MAEs with and without segmentations were statistically analysed using the Wilcoxon signed-rank test. A significance level of α = 0.05 was used.

## Results

### Participants

A total of 82 clinicians participated in this study. They originated from 19 different countries, mostly from the Netherlands (30%), India (16%), France (12%), and the United Kingdom (11%). Of the 82 participants, 75 completed both evaluations with and without segmentation overlays. Seven participants only completed the evaluation without overlays, of whom two did not complete the second evaluation because of technical issues. Each CT was assessed 8–10 times by different observers, of whom at least four were radiologists, both with and without overlaid segmentations. Overall, 66% of the participants were radiologists, 23% were surgeons, and 11% were gynaecologists. The median experience in assessing peritoneal cancer on imaging was 9.5 years (IQR 9.75 years). Regarding the frequency of evaluating peritoneal cancer on imaging, 30% of respondents reported that they evaluated it daily, 56% weekly, 12% monthly, and 1% yearly. Among all participants, 5% always determined rPCI when evaluating peritoneal cancer on imaging, 38% often, 12% sometimes, 20% rarely, and 26% never. For further details on participants’ countries of origin, experience levels, specialists per scan, frequency of assessing peritoneal metastases on imaging, and frequency of assessing PCI on imaging, see S1 Figs in S1 File.

### Interobserver variability

**Fig 2:**
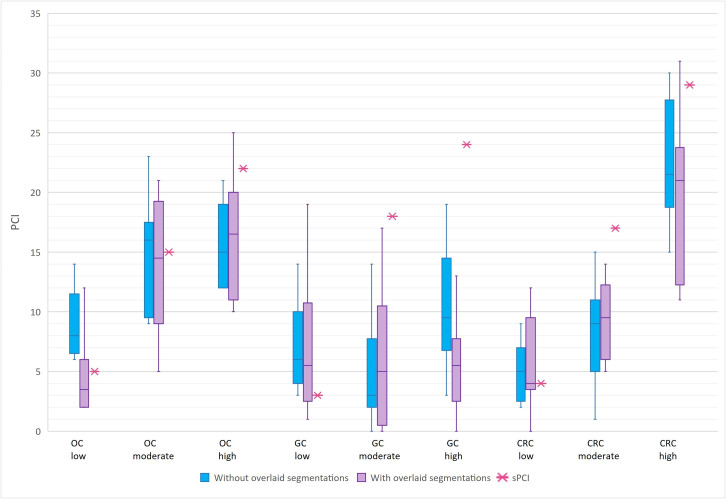
Boxplots of radiological Peritoneal Cancer Index (PCI) scores, without (blue) and with (purple) overlaid segmentations, compared to surgical PCI (pink asterisk). Results are presented across the different CT scans with a low PCI (<10), moderate PCI (10-20) and high PCI (>20) for patients with primary ovarian (OC), gastric (GC) and colorectal (CRC) cancer‌‌.

Fig 2 shows the distribution of total PCI scores per scan, both with and without segmentations used. The IQRs were not lower when using the overlaid segmentations and were even larger in 7/9 of cases. There was no statistically significant difference in IQR (p = 0.123).

### PCI accuracy

In 7 of the 9 scans, the median rPCI was closer to sPCI (pink asterisk, gold standard) with the use of overlaid region segmentations than without (Fig 2). The two scans in which the median rPCI was further from sPCI when using the overlays were the high-PCI gastric and colorectal cases. However, none of these differences were statistically significant (MAE, p = 0.374). Notably, rPCI values were generally underestimated in moderate and high PCI cases, except in the ovarian cancer case with a moderate PCI.

S2 Figs in S2 File present PCI scores stratified by participants’ medical specialty and experience in evaluating peritoneal metastases on imaging. No consistent trends were observed in PCI accuracy or the effect of using region segmentations.

### User’s confidence

The average confidence scores for the 13 regions are visualized in S3 Fig in S3 File. The mean confidence was higher with pre-segmented regions in 6 of the 9 cases and lower in 3 cases (p = 0.593). No significant differences were observed when confidence was analysed per region (p = 0.110) or for individual regions separately (p-values ranged from 0.110 to 0.953; S3 Table in S3 File).

**Fig 3:**
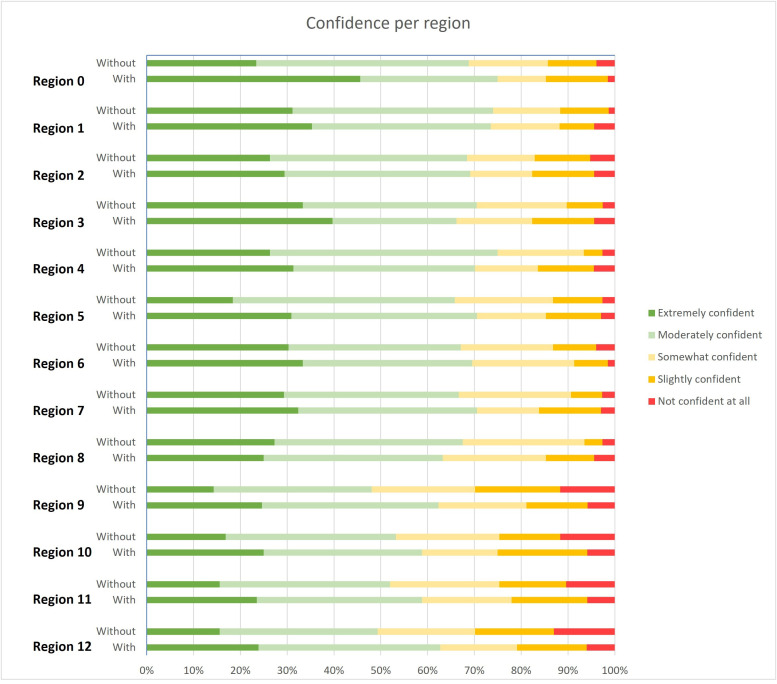
Bar graph showing the level of confidence in radiological Peritoneal Cancer Index (rPCI) assessment per region, with and without colour overlays.

As shown in Fig 3, the proportion of participants reporting extreme confidence was higher when the pre-segmented regions were used, except for region 8. Without overlays, confidence levels were notably lower for regions 9–12, with only 48.1–53.2% of the participants reporting being extremely or moderately confident, compared to 65.8–75.0% for the other regions. With overlays, the proportion of participants with extreme or moderate confidence in regions 9–12 increased to 58.8–62.7%, while changes in the other regions were minor (63.2–75.0%).

### User experience

**Fig 4:**
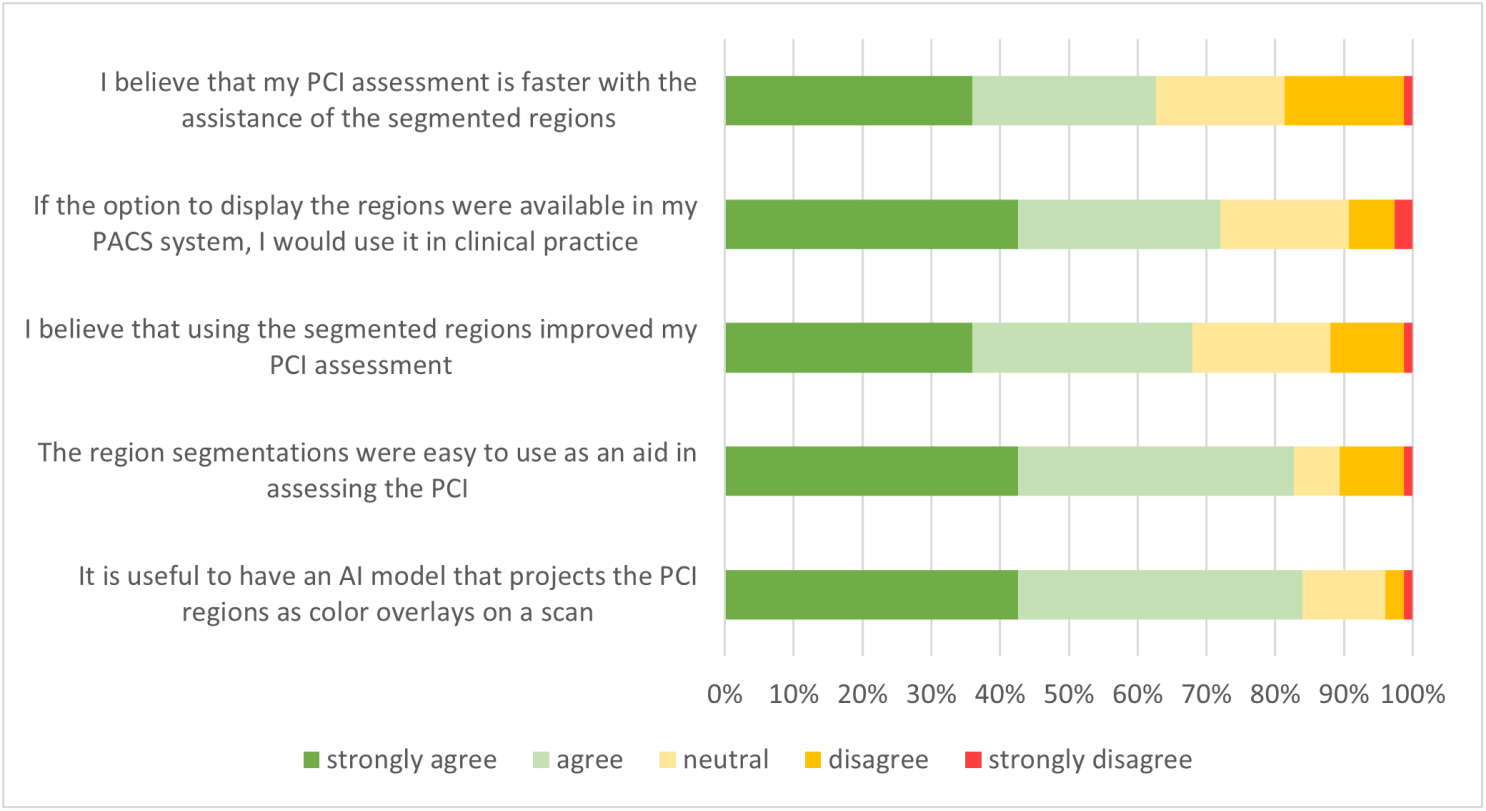
Bar graph showing the participant responses to the five statements regarding the use of pre-segmented PCI regions.

Fig 4 shows participants’ responses to the proposed statements regarding the use of pre-segmented PCI regions. Most participants (83%) agreed that it was useful to have an AI model that projected PCI regions as colour overlays on CT scans. Furthermore, 82% agreed that the region segmentations were easy to use as an aid in assessing rPCI. Of all participants, 67% believed that their rPCI assessments improved by using the segmented regions, and 71% would use the option to display the regions in clinical practice if it was available in their PACS system. Furthermore, 62% of the participants believed that their PCI assessment was faster with the assistance of the segmented regions.

Regarding the measurement of lesion size, 62% reported measuring only in the axial plane, 1% only in the sagittal plane, and 37% measured the largest diameter, regardless of the plane.

## Discussion

In this prospective reader study, pre-segmented region overlays for assessing peritoneal cancer on CT did not significantly reduce interobserver variability, improve PCI accuracy, or increase reported confidence. Nevertheless, several trends were observed: in most scans, the median rPCI was closer to the median sPCI when overlays were used, and more participants reported being extremely or moderately confident, especially in regions 9–12. However, these differences were not statistically significant and should be interpreted with caution. Overall user experience was highly positive, with most participants indicating that overlays were useful, easy to apply, and potentially beneficial for routine practice.

Since the overlays enable clinicians to assess scans region by region in a structured manner, we expected lower interobserver variability in the group using the pre-segmented regions, as detected nodules would be consistently assigned to the same regions. However, interobserver variability was not reduced. One possible explanation is that within each PCI region the score is determined only by the largest visible tumour deposit. If this deposit is clearly located within a region, precise boundary delineation will not improve agreement. Furthermore, most participants were experienced in evaluating peritoneal disease on imaging and may already know the region boundaries quite well, potentially limiting the added value of the overlays. The unfamiliar viewing platform may also have influenced performance. Finally, pre-segmented regions do not resolve the uncertainty whether a structure represents a peritoneal metastasis [21]. Together, these factors may explain why variability was not reduced despite the structured approach. Direct comparison with previous studies is challenging, as most included only two radiologists and reported intraclass correlation coefficients [11]. To our knowledge, this is the first user study investigating the use of colour overlays as a decision aid in PCI assessment.

Although not statistically significant, rPCI accuracy was generally higher with pre-segmented regions, suggesting that a more structured evaluation improves PCI assessment on imaging. Exceptions occurred in gastric and colorectal cases with high PCI, likely because clearer region boundaries do not resolve the challenge of recognizing peritoneal deposits, as discussed earlier. Underestimation was frequent in cases with a moderate or high disease burden, consistent with previous literature [22–24]. This may result from the diffuse spread of small tumour deposits that remain difficult to detect on imaging [5,25–28]. Such underestimation limits the reliability of imaging alone for treatment decision-making. Nevertheless, imaging can complement DLS by guiding surgeons to peritoneal deposits detected on imaging, and potentially avoiding unnecessary procedures when extensive disease is detected.

No clear differences in rPCI accuracy were observed between clinicians’ specialties or experience. These subgroup analyses were descriptive, and no formal statistical comparisons were performed. Given the limited number of cases and the clustered structure of the data, the study was not designed or powered to detect subgroup differences reliably. While greater benefit for less experienced clinicians was expected, the overall high expertise level may have reduced observable differences. Future research including more clinicians could better quantify this effect.

Clinicians’ confidence in assigning PCI scores was generally higher with pre-segmented regions, particularly for regions 9–12 (small bowel). Without overlays, confidence in these regions was lower, consistent with previous literature [14,29]. Increased confidence is favourable, as it suggests greater certainty about assessments; however, it does not necessarily imply improved accuracy. For example, in the gastric cancer case with a high PCI, confidence score was higher with pre-segmented regions (median 4.0 versus 3.5; Fig C1), while accuracy was lower (MAE 18.4 versus 13.6; Fig 2). This illustrates that increased confidence does not necessarily result in better agreement with sPCI. Participants responded positively to using pre-segmented regions for evaluating peritoneal disease on imaging. They generally found the tool useful and indicated willingness to use it in clinical practice. Many participants also perceived a shorter assessment time, although this was not explicitly measured within the study. While perceived usefulness does not necessarily translate into improved diagnostic performance, improved usability may still be relevant for clinical implementation by facilitating a more structured and practical evaluation process. Thus, despite the absence of significant improvements in interobserver variability and PCI accuracy, pre-segmented region overlays may still facilitate the evaluation of peritoneal disease in clinical practice.

This study has some limitations. First, only nine CT scans were used, and the case selection could have influenced the results [21].To reduce this potential bias, a diverse set of cases was selected, including three cases each of ovarian, colorectal and gastric cancer with varying sPCI ranges. Because of the small number of cases, this study should be considered exploratory and was not powered to detect small differences. In addition, multiple readers evaluated the same CT scans, meaning that observations were clustered within cases and not fully independent. Although we used aggregated case-level metrics to partly address this, the hierarchical structure of the data was not fully accounted for. Second, participants had diverse expertise, including radiologists, surgeons, and gynaecologists. The extent to which these medical specialties assess peritoneal disease on imaging varies across countries and centres, but all play a role in this process. Participants differed in experience with evaluating peritoneal disease and PCI on imaging. Although we aimed for a balanced distribution of expertise across scans, variability in participant response made this difficult to achieve. Third, we used CT scans, which is the most widely implemented imaging modality for assessment of peritoneal disease [5,23,28,29]. However, MRI may achieve improved detection of small lesions [5,23,25–28]. Also the emerging technique of fibroblast activation protein inhibitor (FAPI) PET/CT appears promising for assessing peritoneal involvement [30]. Furthermore, we primarily used 3 mm reconstructions to ensure technical stability of the online platform. For detection of small deposits, however, the thin slices could be helpful as well [23]. Future research should therefore investigate other imaging modalities and thin-slice reconstructions.

In this study, manual segmentations were sufficient to address the research aims. For future applications, an AI-based approach is being developed to automatically segment PCI regions on CT, MRI and PET/CT images. Since these regions are based on anatomical structures, the model can be easily adapted across different imaging modalities. Automating region segmentation enables consistent allocation of peritoneal nodules, particularly near region boundaries, and to our knowledge, such an approach has not yet been reported. Moreover, the limited accuracy and frequent underestimation of rPCI compared with sPCI highlight the need for AI tools, not only for region segmentation but also for reliable detection of peritoneal nodules. Most existing AI models have primarily focused on predicting metastases based on the primary tumour, often providing only binary outcomes such as presence or absence, or in some cases a broad classification into low or high PCI, offering limited utility for clinical decision-making [18]. Future work should therefore focus on integrating these AI tools in clinical workflows to ensure clinical added value, through iterative development in collaboration with clinicians and objective evaluation of workflow impact, including reporting time. Additionally, such tools could serve as educational tools, helping less experienced clinicians learn and standardize PCI scoring on imaging. Finally, their clinical utility and diagnostic accuracy should be further evaluated in larger-scale studies to determine their impact on PCI assessment and clinical decision-making.

## Conclusion

Clinicians experienced the use of pre-segmented region overlays for rPCI assessment very positively, describing the tool as useful, easy to use, and clinically valuable. Although no statistically significant improvements were shown in interobserver variability, PCI accuracy, or overall confidence, the positive user experience and the trends observed in this study support further evaluation in larger studies to determine their added clinical value.

## Supporting information

S1 File Supporting Information 1 – User characteristics. (DOCX)

S2 File Supporting Information 2 – PCI accuracy. (DOCX)

S3 File Supporting Information 3 – Confidence. (DOCX)
