# Supplementary material for: Impact of pre-segmented regions on CT-based evaluation of the Peritoneal Cancer Index: A reader study
Source: PLoS One. 2026 Jun 1;21(6):e0349606. doi: 10.1371/journal.pone.0349606 (PMC13225389; doi:10.1371/journal.pone.0349606)
Supplement: S1 File — (DOCX) [file pone.0349606.s001.docx]

Supporting Information 1 – User characteristics


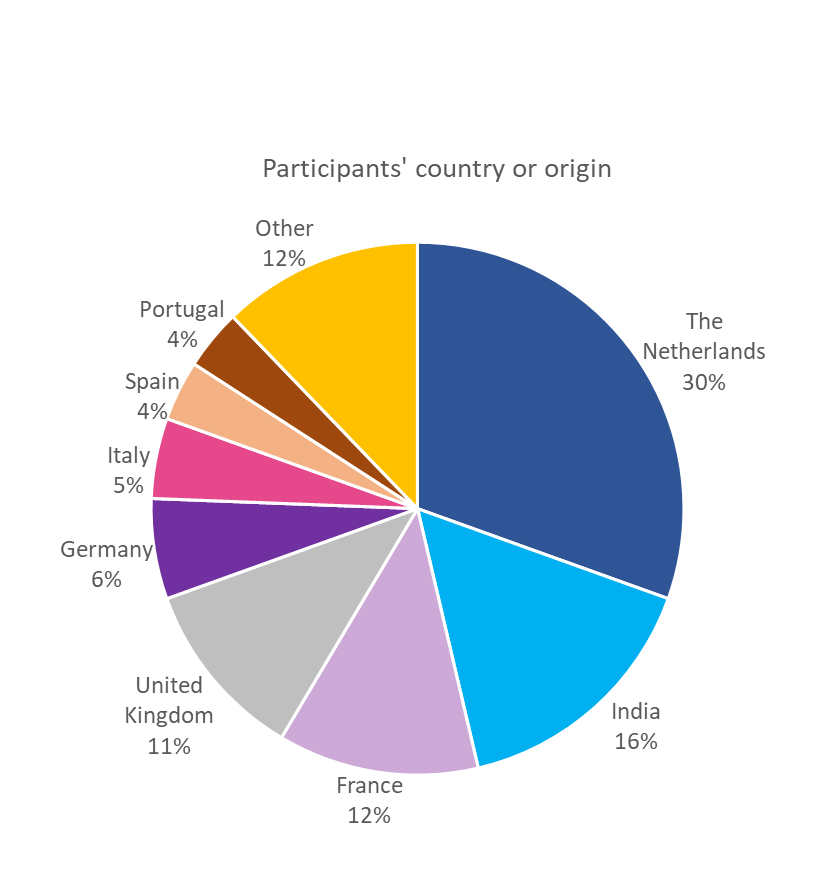


**S1 Fig 1. Pie chart showing the distribution of participants by country of origin.**


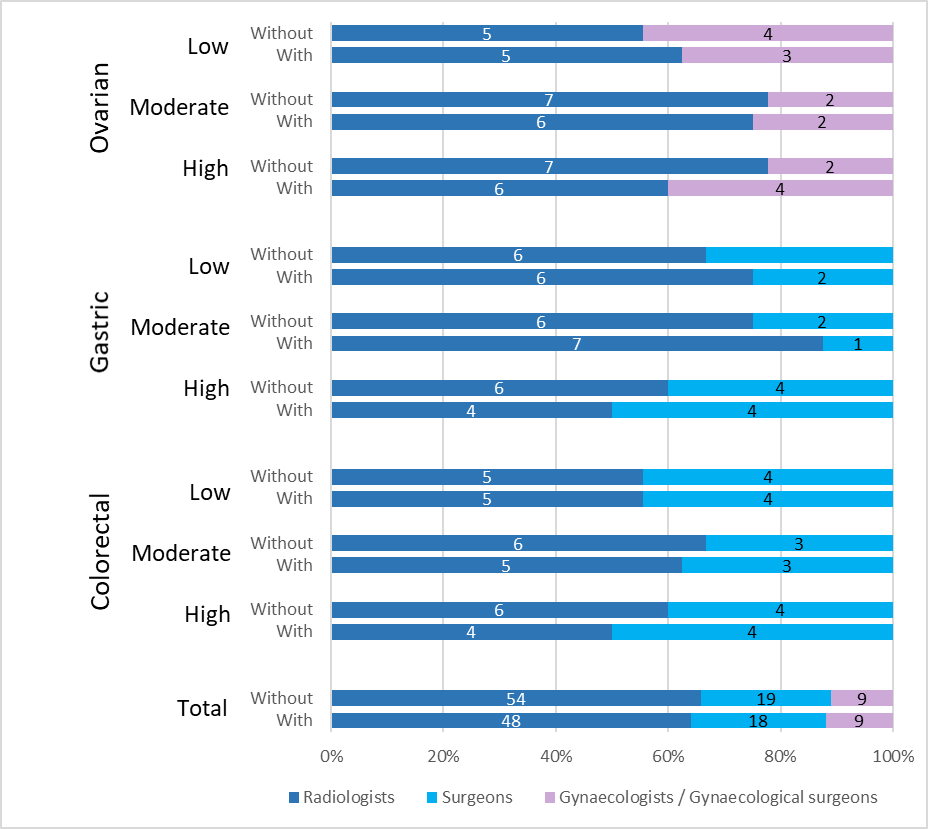


**S1 Fig 2. Bar graph showing the distribution of participants across the different CT scans with a low PCI (<10), moderate PCI (10-20) and high PCI (>20) for patients with primary ovarian, gastric and colorectal cancer.**


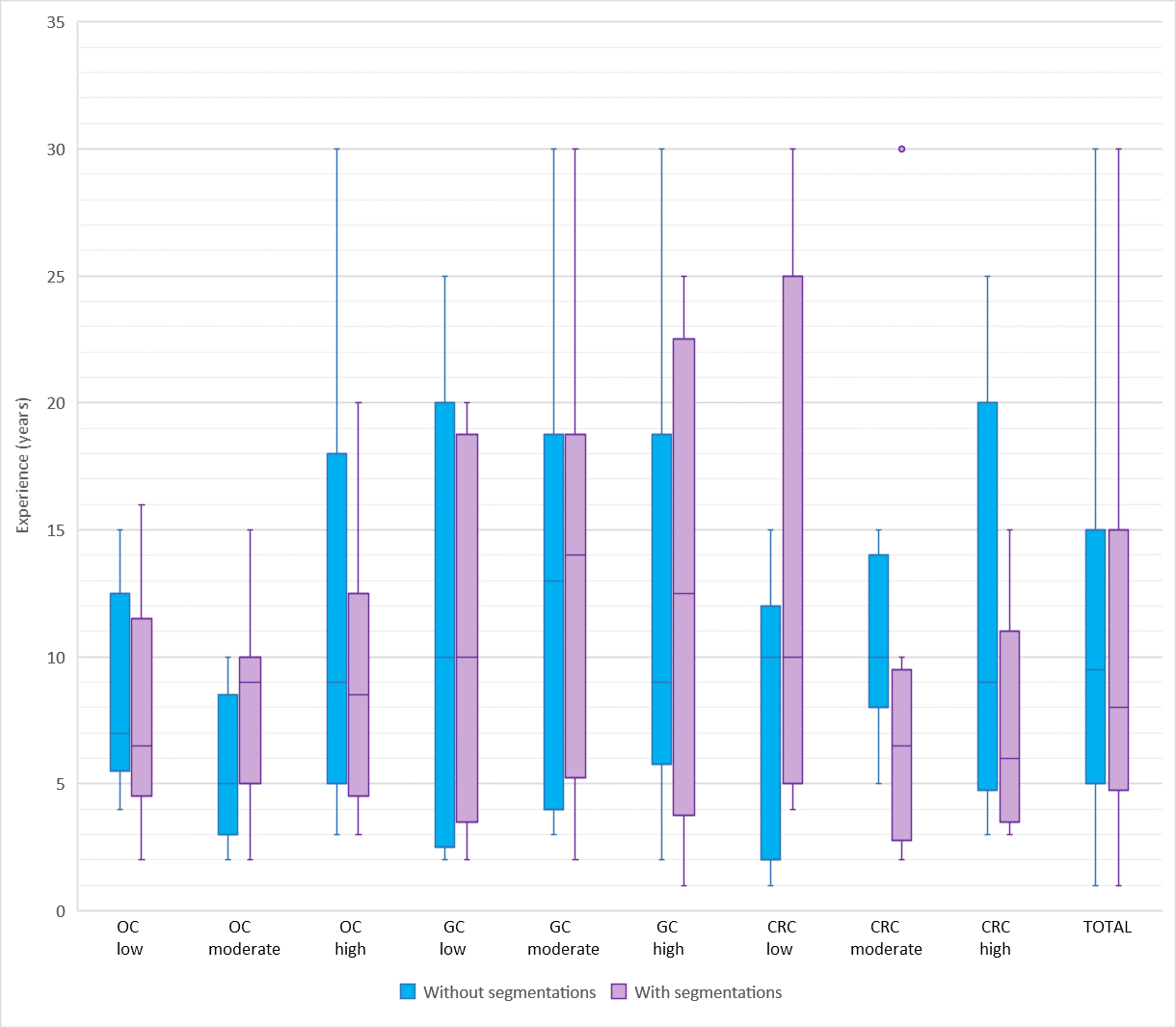


**S1 Fig 3. Boxplots of participants’ years of experience with evaluating the Peritoneal Cancer Index on imaging in the groups with and without the use of the pre-segmented regions across the different CT scans with a low PCI (<10), moderate PCI (10-20) and high PCI (>20) for patients with primary ovarian (OC), gastric (GC) and colorectal (CRC) cancer.**


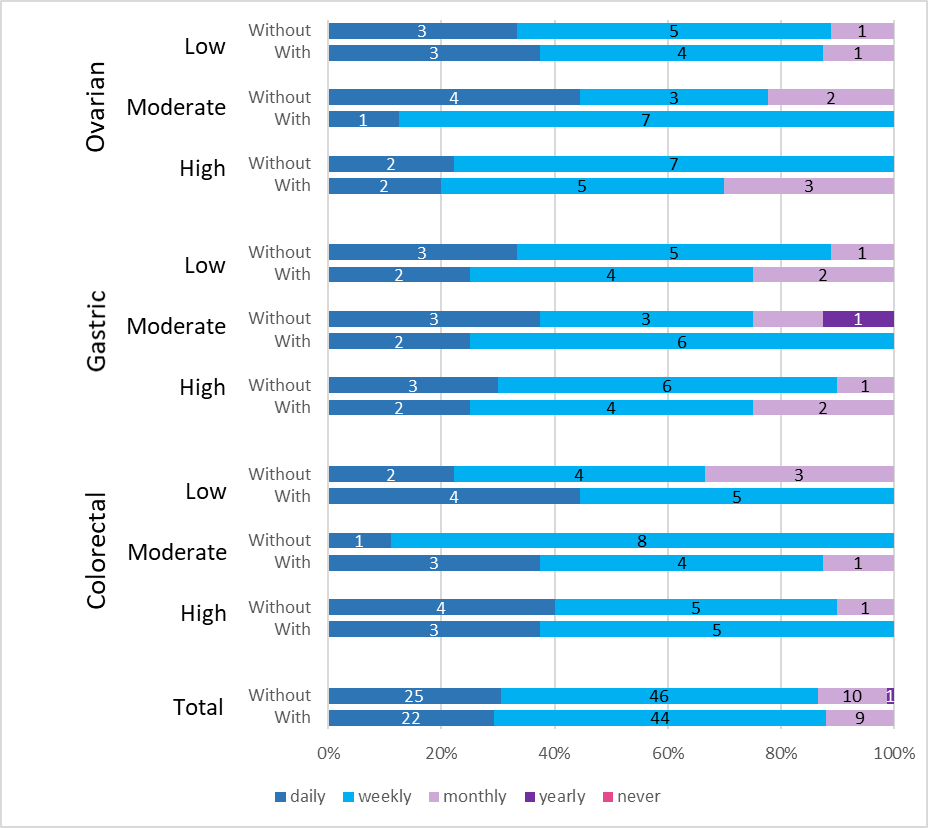


**S1 Fig 4. Bar graph showing participants’ frequency of evaluating peritoneal disease on imaging across the different CT scans with a low PCI (<10), moderate PCI (10-20) and high PCI (>20) for patients with primary ovarian, gastric and colorectal cancer.**


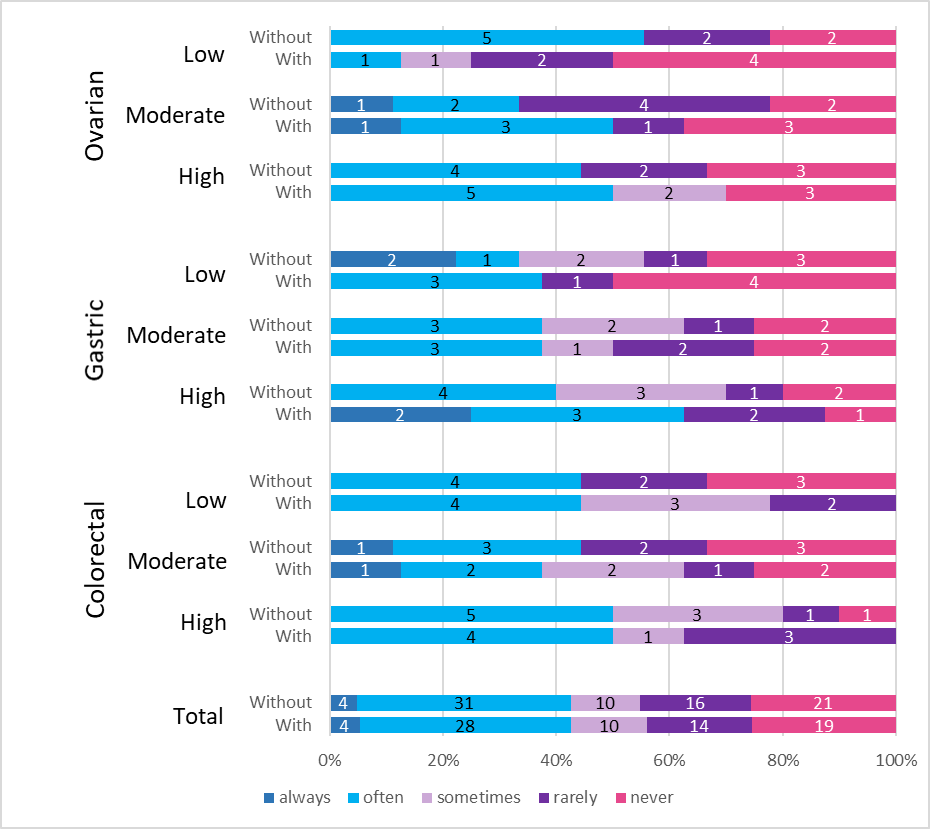


**S1 Fig 5. Bar graph showing participants’ frequency of evaluating the Peritoneal Cancer Index (PCI) on imaging across the different CT scans with a low PCI (<10), moderate PCI (10-20) and high PCI (>20) for patients with primary ovarian, gastric and colorectal cancer.**
