# Supplementary material for: Impact of pre-segmented regions on CT-based evaluation of the Peritoneal Cancer Index: A reader study
Source: PLoS One. 2026 Jun 1;21(6):e0349606. doi: 10.1371/journal.pone.0349606 (PMC13225389; doi:10.1371/journal.pone.0349606)
Supplement: S2 File — (DOCX) [file pone.0349606.s002.docx]

Supporting Information 2 – PCI accuracy


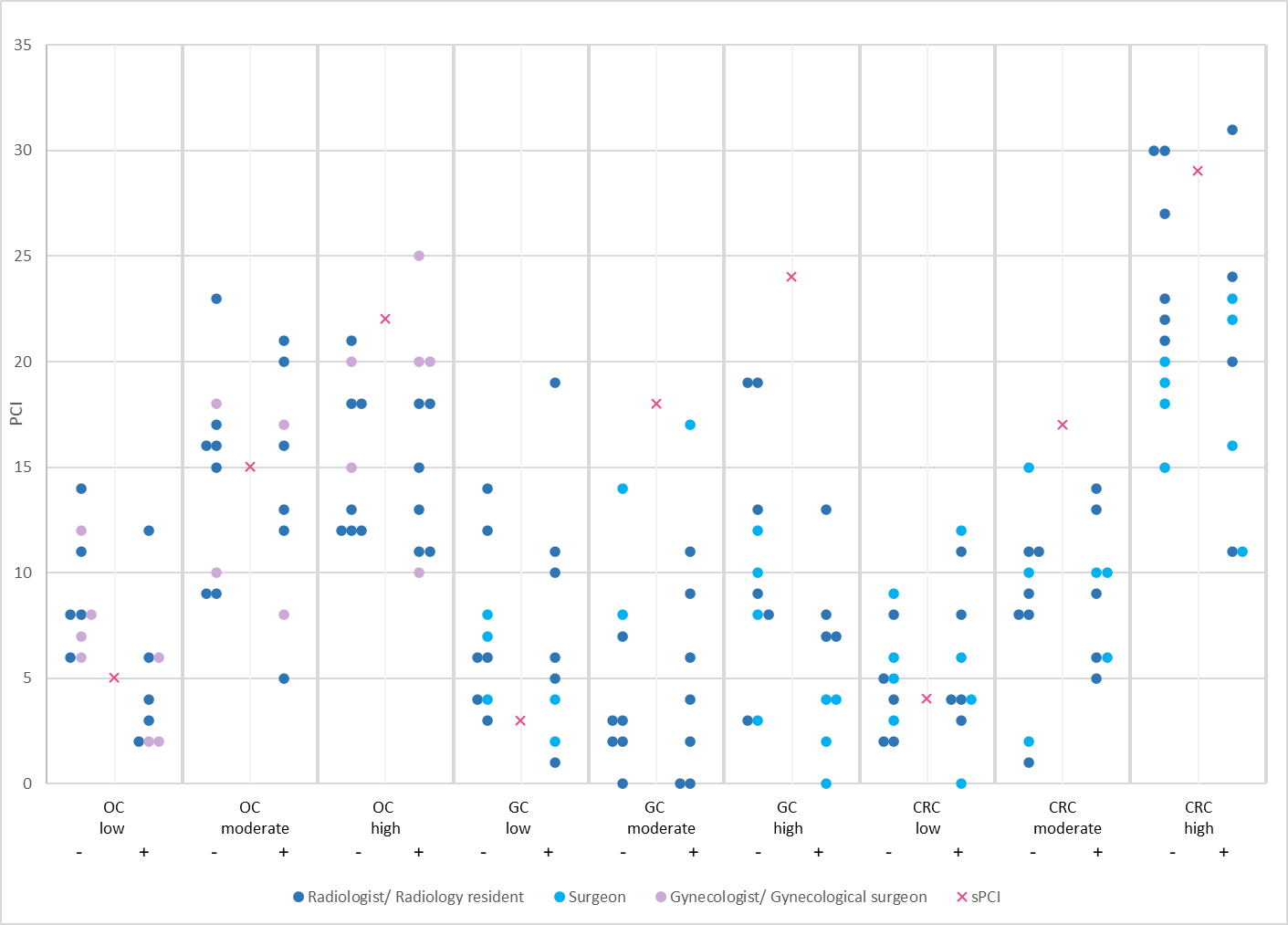


**S2 Fig 1. Scatterplot of the radiological Peritoneal Cancer Index (PCI) on imaging across the different CT scans with a low PCI (<10), moderate PCI (10-20) and high PCI (>20) for patients with primary ovarian (OC), gastric (GC) and colorectal (CRC) cancer, without (-) and with (+) pre-segmented regions overlays, stratified by participants’ medical speciality.**


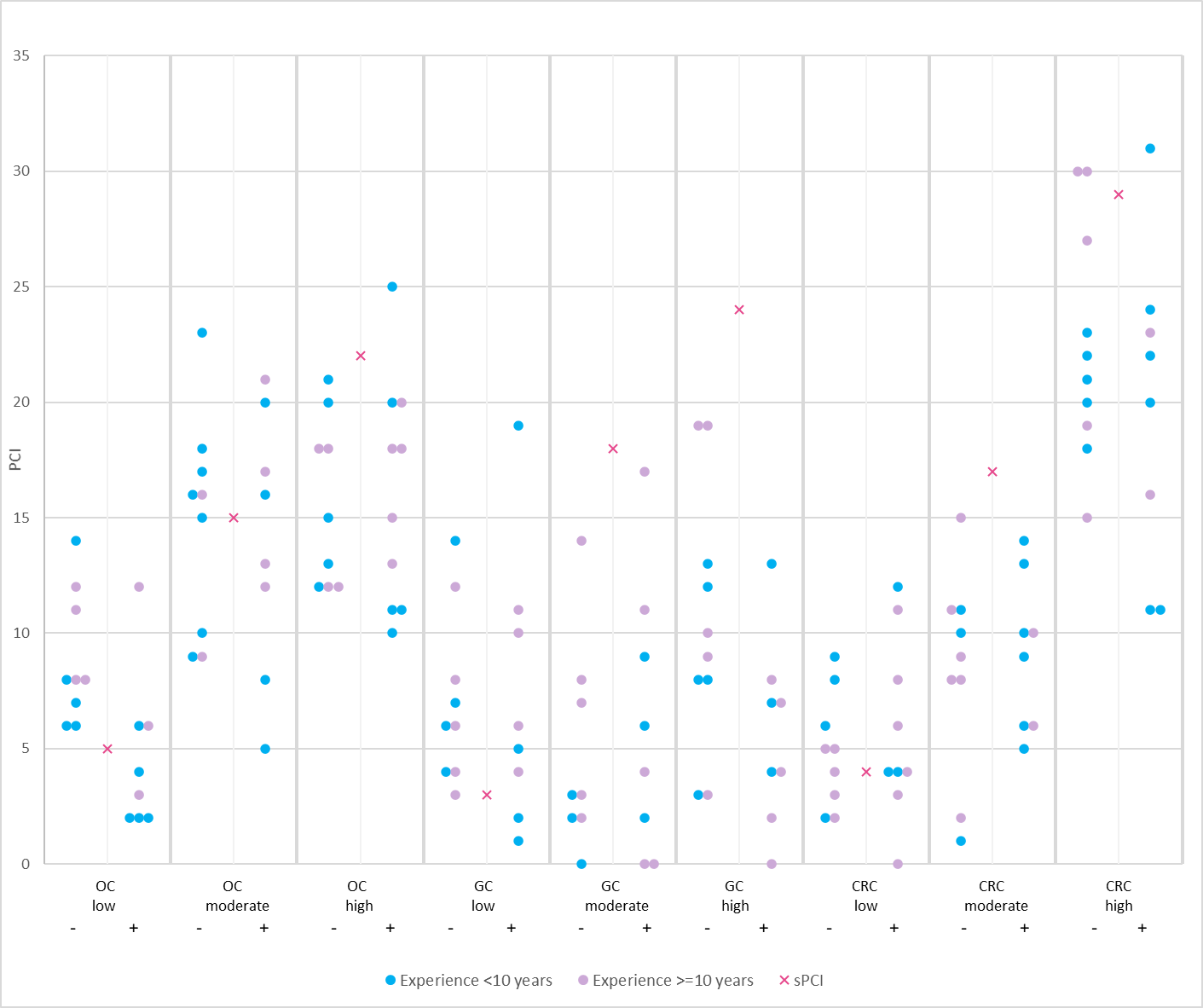


**S2 Fig 2. Scatterplot of the radiological Peritoneal Cancer Index (PCI) on imaging across the different CT scans with a low PCI (<10), moderate PCI (10-20) and high PCI (>20) for patients with primary ovarian (OC), gastric (GC) and colorectal (CRC) cancer, without (-) and with (+) pre-segmented regions overlays, stratified by participants’ experience with evaluating peritoneal cancer on imaging.**
