# Supplementary material for: Impact of pre-segmented regions on CT-based evaluation of the Peritoneal Cancer Index: A reader study
Source: PLoS One. 2026 Jun 1;21(6):e0349606. doi: 10.1371/journal.pone.0349606 (PMC13225389; doi:10.1371/journal.pone.0349606)
Supplement: S3 File — (DOCX) [file pone.0349606.s003.docx]

Supporting Information 3 – Confidence


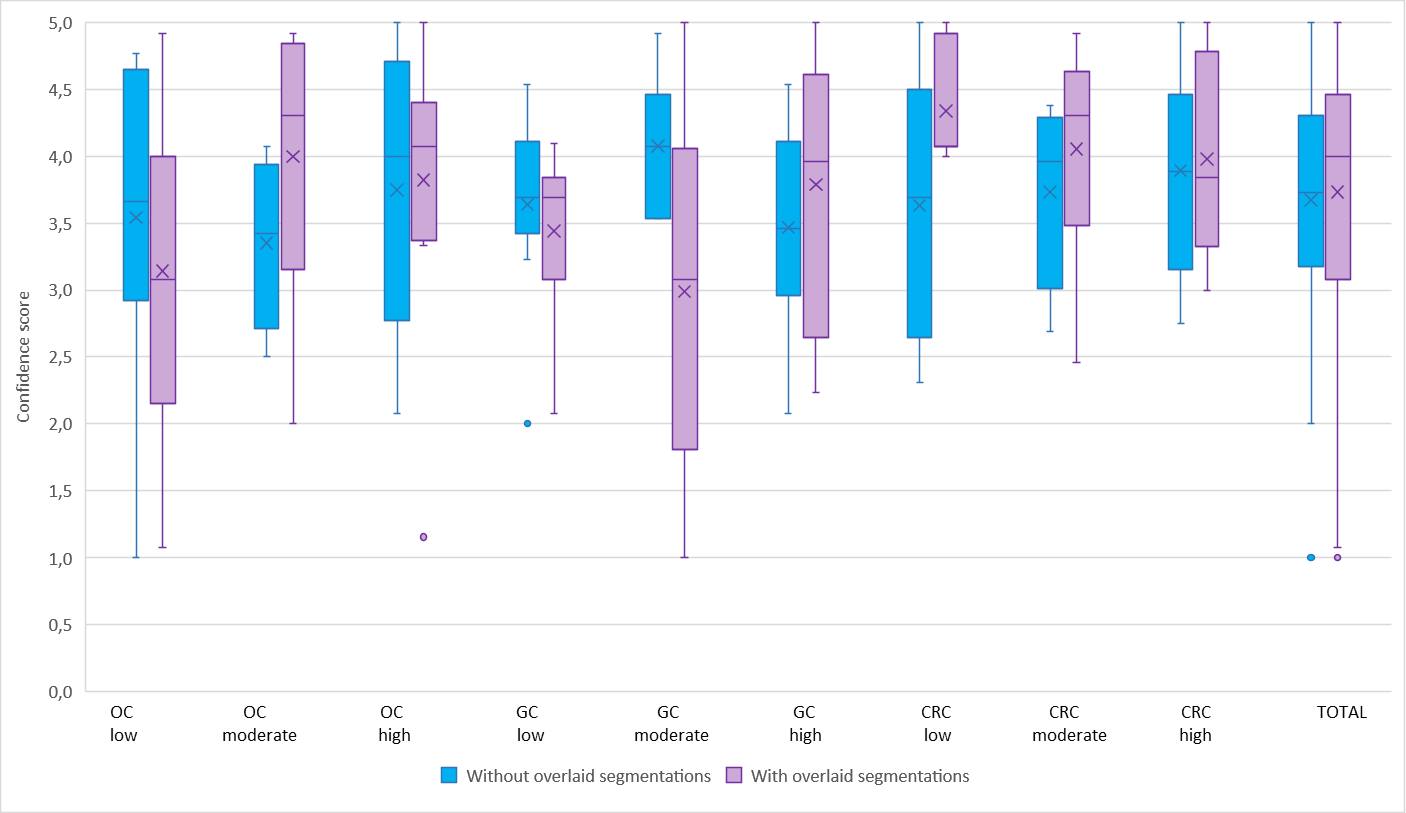


**S3 Fig 1. Boxplots of participant mean confidence scores of all regions (1-5, not confident at all to extremely confident) in the accuracy of their assessment. Scores are shown for the groups with and without the use of the pre-segmented regions across the different CT scans with a low PCI (<10), moderate PCI (10-20) and high PCI (>20) for patients with primary ovarian (OC), gastric (GC) and colorectal (CRC) cancer.**

**S3 Table 1. Mean confidence score compared per region across all scans, without versus with the use of the pre-segmented regions, compared in a paired manner.**

| Region | Mean confidence score without region segmentations | Mean confidence score with region segmentations | p-value |
| --- | --- | --- | --- |
| 0 | 3.7 | 4.1 | 0.214 |
| 1 | 3.8 | 3.9 | 0.594 |
| 2 | 3.7 | 3.8 | 0.953 |
| 3 | 3.9 | 3.9 | 0.779 |
| 4 | 4.0 | 3.9 | 0.441 |
| 5 | 3.7 | 3.9 | 0.484 |
| 6 | 3.8 | 4.0 | 0.678 |
| 7 | 4.0 | 3.9 | 0.314 |
| 8 | 3.9 | 3.7 | 0.374 |
| 9 | 3.3 | 3.7 | 0.123 |
| 10 | 3.4 | 3.5 | 0.515 |
| 11 | 3.4 | 3.6 | 0.286 |
| 12 | 3.3 | 3.7 | 0.110 |
